# Size-Dependent Interactions of Degraded PET Nanoparticles with Human Serum Albumin: Thermodynamic and Molecular Insights

**DOI:** 10.1021/acs.jpcb.5c01362

**Published:** 2025-04-28

**Authors:** Tomasz Panczyk, Pawel Wolski, Krzysztof Nieszporek

**Affiliations:** 1 Jerzy Haber Institute of Catalysis and Surface Chemistry, Polish Academy of Sciences ul, Niezapominajek 8, Cracow 30239, Poland; 2 Department of Theoretical Chemistry, Institute of Chemical Sciences, Faculty of Chemistry, Maria Curie-Sklodowska University in Lublin pl., Maria Curie-Sklodowska 3, Lublin 20031, Poland

## Abstract

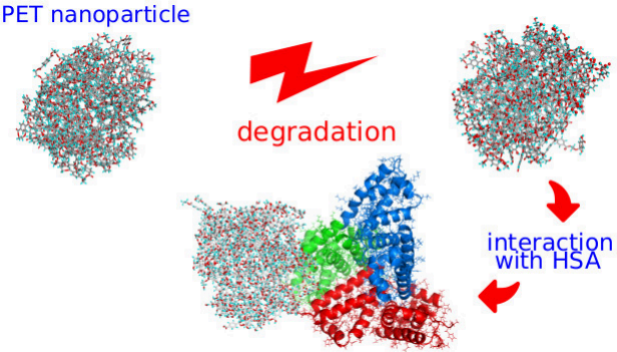

This study examines the interaction between degraded polyethylene terephthalate (PET) nanoparticles and human serum albumin (HSA), focusing on the effects of nanoparticle size and surface modifications resulting from degradation. PET degradation, induced via shock compression in water, leads to significant chemical alterations, including the formation of hydroxyl, carboxyl, and carbonyl groups. These modifications influence the hydrophilicity of PET nanoparticles and their binding behavior with HSA. The production of degraded PET nanoparticles involves subjecting pristine PET to controlled shock compression in an aqueous environment, which initiates chemical reactions similar to those that may occur during degradation. The degradation process is characterized by a progressive breakdown of polymer chains, leading to an increase in functionalized surface groups that enhanced hydrophilicity. The performed analysis of surface chemistry reveals that the introduction of oxygen-containing groups alters the interaction properties of PET nanoparticles, making them more prone to hydrogen bonding with water molecules while simultaneously reducing their affinity for HSA binding. Molecular dynamics simulations, umbrella sampling, and weighted histogram analysis are employed to investigate the thermodynamic aspects of PET-HSA interactions. The study identifies preferred binding sites of PET nanoparticles on HSA, revealing that degraded PET nanoparticles preferentially bind to Domain I and Domain III of HSA. Interaction energy analysis demonstrates that larger PET nanoparticles exhibit stronger binding, whereas small degraded nanoparticles have significantly reduced interaction energies, indicating a higher likelihood of desorption. Further structural analysis using root-mean-squared deviation (RMSD) and root-mean-squared fluctuation (RMSF) confirms that PET binding does not significantly alter HSA’s secondary structure. However, degradation significantly increases PET hydrophilicity, weakening their adsorption onto HSA. Large PET nanoparticles are strongly bound, whereas small degraded nanoparticles remain unbound, raising concerns regarding their potential toxicity due to free migration in the bloodstream. These findings provide crucial insights into the biological implications of PET degradation, the role of surface chemistry in determining nanoparticle interactions, and their potential contributions to nanoplastic toxicity.

## 1 Introduction

Nanoplastics, including those derived from polyethylene terephthalate (PET), pose significant environmental and health risks due to their persistence and ubiquity. These tiny plastic fragments, typically <1 μm in size, are found in various ecosystems, from oceans to drinking water, and their impact on biological systems is an area of growing concern.^1−3^ Nanoplastics have been detected in aquatic environments worldwide, where they are considered a potential threat to both ecosystems and human health. The high surface area and strong binding affinity of nanoplastics enable extensive interactions with surrounding substances, potentially leading to bioaccumulation and toxicity. Upon entering biological systems via ingestion, inhalation, or dermal contact, nanoplastics can interact with proteins and lipids, forming complex structures such as protein coronas. These coronas influence the transport, uptake, and toxicity of nanoplastics, as they modulate interactions with cells and membranes.^1,2^ Moreover, nanoplastics have been implicated in oxidative stress and genotoxicity, particularly when environmental factors like UV radiation alter their properties.^3^

The degradation of macroplastics into nanoplastics occurs via abiotic and biotic processes. Abiotic factors, such as UV radiation, temperature, and mechanical stress, cause polymer chain scission, leading to fragmentation into smaller particles.^4−6^ Abiotic degradation includes photochemical processes driven by sunlight, where reactive oxygen species (e.g., hydroxyl radicals) play a key role in breaking down polymer chains.^7^ Hydrolytic degradation is significant pathway particularly for polymers like PET which hydrolyze into smaller fragments under certain pH conditions.^4^ Typical biotic degradation of plastic involves microbial enzymes breaking down polymer chains into smaller, biodegradable fragments. Enzymes like PETase, produced by the bacterium *Ideonella sakaiensis* accelerate PET degradation, producing intermediates that contribute to further fragmentation.^8^ These processes result in the release of nanoplastics with distinct physical and chemical properties, complicating their environmental detection and impact assessment.

Human serum albumin is the most extensively studied serum protein. It is a nonglycosylated globular protein with a molecular weight of 66 748 kDa, comprising 585 amino acid residues and 17 disulfide bridges. Its structure is well-characterized, featuring three homologous α-helical domains with two distinct ligand-binding sites located in hydrophobic cavities of subdomains IIA and IIIA, known as site I and site II, respectively. HSA plays a crucial role in transporting nutrients and small molecules within the body. Due to its constant exposure to foreign particles entering the circulatory system, HSA can interact with these substances.^9,10^ These nonspecific interactions can lead to structural and functional alterations in HSA, potentially causing denaturation and loss of its biological activity.^11^ Therefore, investigating the interaction between microplastics/nanoplastics and HSA is critical for understanding the potential detrimental effects on HSA’s function and its biological activity in vivo.

Nanoplastics can interact with biological macromolecules, forming protein coronas that significantly alter their behavior in living systems. Studies reveal that the surface modifications and size of nanoplastics influence the structure and dynamics of protein coronas, affecting their biological activity and toxicity. For example, polystyrene nanoplastics form both “hard” and “soft” coronas with HSA, depending on factors like nanoparticle size and pH.^12^ These interactions can lead to protein denaturation and altered signaling pathways.^12^ Moreover, specific proteins in the corona, such as immunoglobulins or enzymes, may become denatured upon binding, leading to loss of function or unintended immune responses.^1^ Several recent studies have investigated the interaction between nanoplastics and biological interfaces. These studies have revealed the presence of various proteins within the corona, including HSA, lysozyme, fibrinogen, and immunoglobulins.^13,14^ The interplay between nanoparticles and proteins is complex and reciprocal. Proteins can significantly alter the surface properties and biological pathways of nanoparticles. Conversely, nanoparticles can influence the structure and function of proteins, as observed with amylin,^15^ hemoglobin,^16^ insulin^17^ and albumin.^18^

A variety of advanced analytical techniques have been developed to characterize nanoplastics and their interactions with biologically active molecules. Raman and infrared spectroscopy are frequently employed to identify the chemical composition and surface properties of nanoplastics. By analyzing the vibrational modes of molecular bonds, it is possible to determine the extent of degradation and functionalization of nanoplastics.^19^ Atomic force microscopy (AFM) and scanning electron microscopy (SEM) allow the visualization of nanoplastic morphology and surface roughness, providing detailed insights into structural changes induced by environmental interactions.^20^ Dynamic light scattering (DLS) measures the size distribution and aggregation behavior of nanoplastics in different solution chemistries, including varying ionic strengths and pH levels.^21^ Fluorescence and Circular Dichroism Spectroscopy are critical for investigating how nanoplastics interact with proteins. Changes in protein conformation, such as unfolding or denaturation, can be detected, providing insights into how protein coronas form on nanoplastics and influence their biological activity.^12,19,21^ Analysis of adsorption models is also applied to study how proteins or other biomolecules adhere to nanoplastics. They provide quantitative descriptions of binding affinities and capacities, particularly for nanoplastics with different surface modifications.^2^

Molecular simulation is a powerful tool for studying phenomena at the nanometric scale. It has proven remarkably successful in predicting macroscopic thermodynamic and dynamic observables for a wide range of systems. Moreover, it is increasingly becoming a valuable option for describing system properties under conditions where experimental determinations are challenging to obtain.^22^ Recent work by Zhang et al.^23^ provides insights into the molecular interactions and aggregation mechanisms of several types of nanoparticles. They studied aggregation and interactions with humic acid moieties using pristine and aged small polymer clusters. The aging/degradation was simulated by altering the chemical composition of polyethylene, polyvinyl chloride, and polystyrene. This was done by inserting carboxyl, hydroxyl, and carbonyl groups into the initial molecular structures of these polymers. They found aggregation of pristine nanoparticles due to hydrophobic interactions and attachment of humic acid particles to aged nanoparticles due to the cation bridging effect.

Hollóczki and Gehrke^24^ demonstrated through simulations that a phospholipid membrane undergoes significant readjustment in the presence of polyethylene nanoparticles with a diameter of approximately 5 nm. This finding suggests that the presence of nanoparticles has a substantial effect on biological membranes. Bochicchio et al.^25^ conducted a study utilizing coarse-grained simulations of polyethylene, polystyrene, and polypropylene interacting with phospholipid membranes. All nanoparticles rapidly entered the membrane and exhibited remarkable differences in the behavior of the three polymers once inside lipid bilayers: PE showed a strong tendency to self-aggregate, forming lens-shaped clusters, while PS and PP dissolved completely within the membrane. Hollóczki and Gehrke^26^ investigated the interactions of four types of nanoplastic particles with a series of proteins. Their findings revealed that amino acid polarity is a crucial factor influencing the adsorption of proteins onto nanoparticles. Hollóczki^27^ employed simulated annealing molecular dynamics to generate and study an array of conformations for a sample oligoalanine peptide binding to polyethylene and nylon-6,6 nanoplastics. The resulting structures, with diameters up to 5 nm, were further investigated using static quantum chemical calculations. The obtained data unequivocally demonstrate that both plastic nanoparticles exert a strong influence on the relative stability of α-helix, β-hairpin, and other conformations.

The degradation mechanisms of several plastic materials — LDPE (low-density polyethylene),^28^ PET,^29^ polypropylene^30^ and polystyrene^30^ — have been thoroughly studied by Panczyk et al. using reactive molecular dynamics. The application of reactive force fields (ReaxFF)^31−33^ aimed to directly track chemical transformations occurring in these materials when subjected to either mechanical stress or chemical activation. Mechanical stress was used to analyze surface structures formed upon cleavage, while chemical activation was applied to identify the most likely chemical reactions occurring in the material during prolonged exposure to UV radiation. The study revealed that each material has its own distinct ″chemistry,″ developing unique surface functional groups with complex chemical linkages and radicals. However, these studies were conducted under somewhat unrealistic conditions, as water and oxygen were not included in the simulation boxes.

Modeling chemical activation of reactions in molecular dynamics simulations is not straightforward. One approach is to heat the sample; however, achieving sufficient acceleration of inherently slow reactions would require extremely high temperatures, leading to melting or other significant changes in the material’s physical properties. It has been demonstrated that shock compression of polymers can initiate reactions without significantly altering the sample structure (at least under moderate compression pressures).^30,34,35^ Shock compression in molecular simulations is a powerful tool for exploring material behavior under extreme pressure and temperature conditions, such as those encountered during impacts, explosions, or within planetary interiors. This technique provides insights into how a material’s structure, dynamics, and thermodynamic properties evolve when subjected to a sudden, high-pressure shock wave. Molecular simulations utilize these principles to study atomic-scale phenomena that are often difficult to observe experimentally. The methodology is based on the Rankine-Hugoniot relations,^36^ which are derived from the fundamental conservation laws of mass, momentum, and energy across a shock front. These relations establish a link between the initial and final states of a material, describing changes in properties such as pressure, density, and internal energy following shock compression.

In this study, we propose a novel approach to analyzing the interaction between degraded PET nanoparticles and human serum albumin. Special attention will be given to the chemical composition of degraded plastic nanoparticles, as the applied methodology—conducting degradation reactions in an aqueous medium using ReaxFF and shock compression—results in substantial and chemically relevant modifications of the PET surface. To assess whether PET weathering enhances or reduces interactions with HSA, we will investigate the adsorption behavior of both degraded and intact PET nanoparticles on the protein surface. Addressing this problem requires the development of a dedicated methodology, as fully exploring the phase space for such large molecules in molecular dynamics simulations remains a challenge. The potential toxicity of degraded PET nanoparticles in humans will be evaluated based on the extent of interaction, including binding energy, binding sites, the reversibility of the binding process, and structural alterations in the protein. Therefore, this research focuses on identifying the binding patterns, key interaction sites, and forces responsible for perturbing the conformational stability of HSA at the molecular level, complemented by a thermodynamic analysis.

## 2 Methods

### 2.1 Construction of the Molecular Model of Intact PET Nanoparticles

The study required an atomic-level representation of polyethylene terephthalate and the application of a suitable force field to describe its dynamics in the unaltered chemical state. The molecular model was initiated by manually generating a single ethylene terephthalate monomer. This monomer was then replicated to create a polymer chain consisting of 100 repeating units, resulting in a molecular mass of 19 246 g/mol, which is representative of typical PET materials.^37^ The replication of the monomer (polymerization) was performed using the tleap program from the AmberTools package.^38^ This program requires selecting an appropriate force field for the molecule being processed. For this purpose, we utilized the AMBER force field,^39^ and the partial atomic charges were calculated using the RESP procedure.^40^ The polymerization performed using tleap produced long linear chains. To obtain roughly spherical and larger PET nanoparticles, we combined two or four such linear chains and conducted molecular dynamics simulations in the NVT ensemble without water. The chains quickly folded into approximately spherical nanoparticles.

### 2.2 Equilibration of PET Nanoparticles in the Presence of Water

The obtained atomic structures of the PET nanoparticles were used as input for constructing simulation boxes for molecular dynamics simulations with the ReaxFF force field.^31,33^ This force field was selected because the hydrolytic degradation of polymers involves chemical reactions, and in the specific case of PET, three different elements participate in these reactions. Additionally, the nanometer scale of the system under investigation effectively rules out the use of quantum chemical methods, such as density functional theory, due to their high computational cost.

The ReaxFF force field belongs to a class of reactive force fields, distinguished by its bond-order-dependent potential. This framework inherently incorporates van der Waals and Coulomb forces and derives dissociation and reaction curves based on quantum chemical calculations. The total energy of the system is determined by a complex combination of components, including interatomic distances, bond orders, atomic coordination (both over- and under-coordination), lone pair energies, valence angle terms, torsions, and various penalty terms related to angles and bonds. It also accounts for conjugation energy in aromatic systems, van der Waals interactions modeled using the Morse potential, and Coulomb interactions. Atomic charges in ReaxFF are computed using a geometry-dependent charge calculation scheme, utilizing either the electronegativity equalization method (EEM)^41^ or the Qeq scheme.^42^

ReaxFF has undergone extensive parametrization for various combinations of elements and conditions, making it an exceptionally versatile tool for modeling chemical systems. Nevertheless, the application of ReaxFF requires a careful analysis of the studied system when choosing an appropriate parameter set. In the specific case of PET reactions with water, the recent parametrization for the description of functionalized hydrocarbon/water weak interactions in the condensed phase (CHON-2017_weak)^33^ is the most suitable. The validation of ReaxFF in the degradation reactions of PET has already been done in the case of mechanical cleavage of bulk material^29^ and the comparison of the obtained results with the literature data published by Gewert et al.^4^ Thus, the equilibration stage was performed using the aforementioned force field parameter file, with the atomic coordinates of PET obtained from simulations with the nonreactive AMBER force field, as previously mentioned. The PET nanoparticles were positioned at the center of a cubic box with dimensions of 10 × 10 × 10 nm^3^ or 9 × 9 × 9 nm^3^, and the remaining space was filled with water using a custom-designed script. The geometry of the water molecule was taken directly from the TIP3P water model, and the number of inserted molecules was 13 675 or 10 131 for the larger or smaller nanoparticle, respectively. The equilibration runs were carried out in the NPT ensemble with the temperature and pressure set to 300 K and 1 bar, respectively. The applied barostat was Nosé-Hoover with time constants for temperature and pressure coupling of 50 and 500 fs, respectively. The integration time step was 0.25 fs, and the total equilibration runs encompassed 1 million timesteps. The partial charges on atoms were determined using the charge equilibration scheme Qeq^42^ The final states of the simulation boxes, i.e., PET nanoparticles surrounded by water, are illustrated in the Supplementary File in Figure S1. The sizes of the nanoparticles, expressed as gyration radii, were 2.31 and 1.82 nm, depending on the initial number of PET chains used.

### 2.3 Degradation of PET Nanoparticles via Shock Compression

The degradation of plastic nanoparticles consists of a set of chemical reactions that depend on the chemical environment of the participating atoms and the energy input required to overcome activation barriers. This phenomenon, including hydrolytic degradation, is a slow process, as plastics persist in water or soil as contaminants for many years.^4,5^ Observing such reactions within a simulation box over a time scale of just a few nanoseconds is, therefore, highly improbable. Consequently, some means of accelerating these reactions is necessary in computational studies. One approach is to use elevated temperatures to overcome activation barriers and initiate reactions. However, as we found in preliminary studies, high temperatures alone are not very effective, as they lead to undesirable phenomena such as polymer melting and do little to initiate chemical reactions. Instead, the combination of high temperatures and pressures, applied simultaneously in the form of a shock wave passing through the sample, proved to be a highly effective method for controllably modeling the initiation of chemical reactions.^28,30^

The shock wave passage was modeled using LAMMPS implementation of the Rankine-Hugoniot relations, that is fix nphug.^43^ Instead of directly inducing a shock wave, the fix adjusts system parameters to satisfy the Hugoniot conditions, creating a virtual shock that corresponds to a specific pressure and temperature state.^36^ The key parameter that needs adjustment is the maximum pressure to which the system is compressed as a result of the shock wave passage. By increasing the compression pressure, the system experiences higher temperature and pressure, which can, in turn, initiate chemical reactions in addition to physical (typically reversible) deformation of the compressed material.

**Figure 1:**
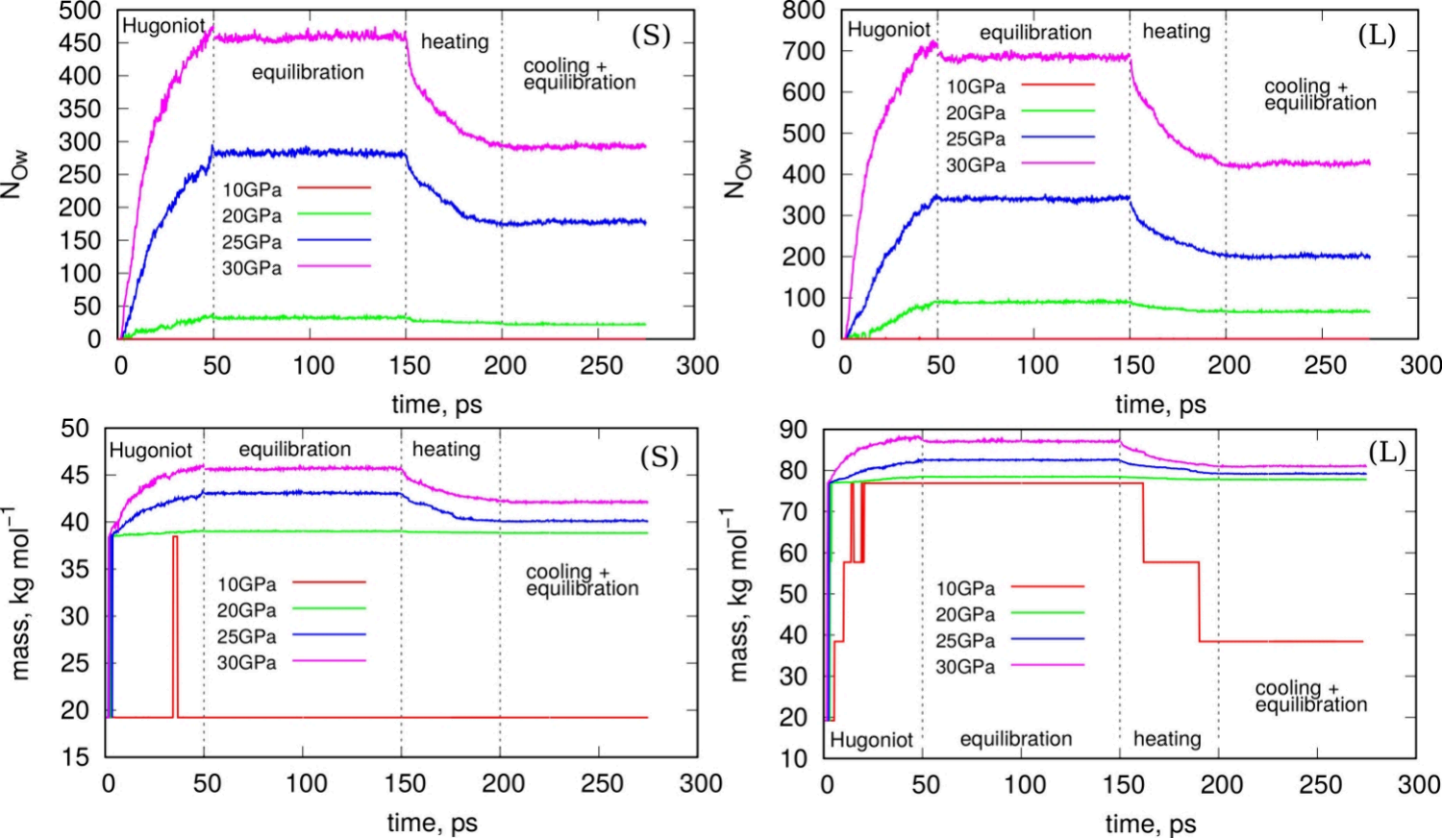
Illustration of the degradation of PET nanoparticles in water due to the application of shock compression. The upper panel shows the increase in the number of oxygen atoms added from water over time and as a function of compression pressure for small (S) and large (L) nanoparticles. The bottom panel depicts how the mass of the heaviest component changes over time for various compression pressures and for small (S) and large (L) nanoparticles. The dashed vertical lines indicate changes in the sample treatments. The range labeled “Hugoniot” corresponds to the stage of shock compression of the samples, with the compression pressures indicated in the legend. The next stage, “equilibration”, refers to normal dynamics in the NPT ensemble with the temperature set to 300 K. The subsequent stage, “heating”, involves heating the samples to 1000 K in the NVT ensemble to break transient bonds. Finally, the “cooling + equilibration” stage involves cooling the samples to 300 K and performing normal dynamics in the NPT ensemble with the pressure and temperature set to 1 bar and 300 K, respectively.

The studied samples (small and large PET nanoparticles surrounded by water) were subjected to four increasing compression pressures: 10, 20, 25, and 30 GPa. Initially, we used evenly spaced values of 10, 20, and 30 GPa and found that at 30 GPa, significant material destruction occurred, indicating that this value is too high to ensure the gradual activation of reactions from the most to the least prone. On the other hand, 20 GPa led to only minor surface modifications. Therefore, we introduced an additional compression pressure of 25 GPa, which proved to be well-suited for the gradual activation of PET reactions with water. The applied pressures led to specific behaviors: at 10 GPa, oxygen atoms from water still have not attached to the PET surface; at 20 GPa, only a few oxygen atoms attached; and at 25 and 30 GPa, the PET surface has been significantly modified by the attachment of several hundred oxygen-containing groups. Hugoniostat dynamics simulations were performed for 50 ps for each system, followed by an additional 100 ps of relaxation in the NPT ensemble. The systems were then annealed at high temperature to break weakly bonded species and subsequently cooled to normal temperature. These treatments are summarized in Figure 1 and visualizations of their atomic structures are shown in Figure S2. The PET nanoparticles obtained after 25 GPa compression pressure were used in subsequent studies as models of degraded nanoplastic particles.

### 2.4 Generation of AMBER Force Field Parameters for Degraded PET Nanoparticles

The study of interactions between degraded PET nanoparticles and other organic molecules, particularly HSA, requires the generation of pairwise additive force field parameters. Typically, within the AMBER force field, new and nonstandard molecules are described using the General AMBER Force Field (GAFF).^39^ Thus, we adopted this approach, although it required a detailed analysis of the degraded PET nanoparticles in the context of their bond topology.

These nanoparticles are too large to be processed by automated tools such as antechamber, parmchk, etc., which are distributed within the AmberTools package. Therefore, we designed a dedicated code that analyzes each atom in the nanoparticles. Depending on its chemical environment, the number of bonds, and the angles associated with it, the code assigns an appropriate atom type from the GAFF atom type database. Additionally, we utilized atomic charge values obtained from the charge equilibration procedure required by the ReaxFF force field. These charges were output at specific integration timesteps along with the complete bond topology during the ReaxFF simulations.

By assigning GAFF atom types and partial charges to each atom in the degraded PET nanoparticles, we were able to generate the full AMBER force field parameter set using tleap from the AmberTools package.^38^ The generated parameters were then converted into the GROMACS format. The force field files in GROMACS format are available in the Supporting Information.

### 2.5 Exploring the Configurational Phase Space of PET-HSA Interactions

Finding an optimal binding configuration of two large objects in all-atom molecular dynamics simulations is challenging, as the configurational phase space is typically filled with local minima, leading to the system becoming trapped in metastable yet long-lasting configurations. To address this issue and make the problem more tractable, we adopted a specialized methodology derived from the rigid body replica exchange method, originally developed for studying interactions between carbon nanotubes and telomeric DNA.^44^

In the first step, the studied PET nanoparticle and HSA were placed in a single simulation box at the shortest possible distance. The initial conformation of HSA was retrieved from the Protein Data Bank (PDB ID: 1N5U(45)), and its protonation state, corresponding to physiological pH = 7.4, was determined using the H++ web service.^46,47^ For further studies, HSA was modeled using the AMBER ff14SB^48^ force field, including partial charges. The PET nanoparticle structures were taken from the final simulation frames, either from intact nanoparticles described by the AMBER force field or from the last simulation frames obtained in a series of simulations corresponding to shock compression and relaxation. The force field used for degraded PET nanoparticles was the general AMBER force field (GAFF)^39,49^

To estimate the potential localization sites of PET on the HSA surface, we employed a search process based on an implicit-solvent rigid-body motion method. The implicit solvent model significantly accelerates calculations, while the rigid-body representation of both HSA and PET prevents deformation of these species in a vacuum. All Lennard-Jones energy parameters in this approach were scaled by a factor of 0.1 to smooth the potential energy surface, allowing the system to escape from local energy minima and enhancing the sampling of less energetically favorable regions. We applied a simple implicit solvent model based on Debye screening of electrostatic interactions, using the following formula for the Coulomb interaction energy between two point charges:
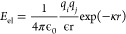
1where *E*_*el*_ is Coulombic pairwise interaction energy between point charges *q*_*i*_ and *q*_*j*_, ε is the relative permittivity of water, ε_*0*_ is permittivity of free space, *r* is is the distance between the point charges, and κ is the Debye screening length in an electrolyte with an 0.15 mol L^–1^ ionic strength.

The motion of the PET nanoparticle on the HSA molecule surface, i.e., scanning the possible binding sites, was enforced by applying the enhanced sampling method known as metadynamics.^50^ In this method, a set of collective variables is used to bias the motion against local energy barriers by smoothing the potential energy surface through the addition of Gaussian hills to the system’s Hamiltonian. In this particular case, the collective variables were chosen so that the PET nanoparticle was forced to sample the polar angle, measured from the *z*-axis with respect to the HSA molecule center of mass, and the azimuthal angle of its orthogonal projection onto the x–y plane. Additionally, the distance between the centers of mass of PET and HSA was restricted to 7 nm, by applying a reflecting wall, to prevent exploration of conformational space far from the HSA surface. With this selection of collective variables and restraints, the PET nanoparticle was forced to roll across the surface of HSA, and over time, it gained an increasing ability to jump over local potential barriers. This was due to Gaussian hills, each with a height of 0.8 kJ/mol, being deposited every 1000 steps, and the Gaussian functions being rescaled using a bias factor of 20. For all models, the metadynamics simulations were conducted for 6.8 to 9 millions of steps. All metadynamics simulations were performed using LAMMPS^51^ patched with PLUMED 2.6.^52^Figures S3 and S4 show the rescaled binding energies and representation of the trajectories obtained for all the studied cases.

In this way, we quickly obtained a long trajectory of many different configurations of PET on the HSA surface, which corresponded to scanning the configurational space, though with the interaction energies strongly influenced by the energy scaling factors applied in the metadynamics simulations. Therefore, in order to recover the true energy landscape, still limited to HSA-PET interactions only (without explicit water), the obtained trajectory was supplied to the GROMACS^53^gmx_energy module, which calculated the interaction energies corresponding to the configurations taken from the previous calculation step. In this way, we obtained a set of configuration-energy pairs and selected the lowest energy one for further analysis, (Figure S3). The lowest-energy configurations were used as the starting configuration for all-atom calculations with explicit water.

Thus, the core of the above method is the rapid scanning of potential binding interaction configurations, primarily using geometric matching as the decisive criterion. These configurations serve as starting points for a full dynamic simulation, allowing for flexible binding interactions. Thus, a potential limitation of the method arises when geometrically weakly matched species end up in the best-matched configurations after the flexible dynamic stage. However, this is rather an unlikely scenario.

### 2.6 Equilibrium and Umbrella Sampling Computations

The lowest energy configurations of the HSA-PET systems, determined through the search procedure described in the previous subsection, were placed in a periodic box of a size ensuring adequate space between system components. The details of the simulation box sizes and compositions are provided in Table S2. Next, the boxes were filled with TIP3P water molecules and neutralized by adding counterions and additional ions to achieve a concentration of 0.15 M NaCl. To remove steric clashes between the water molecules and the solute, we performed energy minimization for 10 000 steps using the steepest gradient method. The systems were equilibrated in both the canonical (NVT) and isobaric–isothermal (NPT) ensembles for 1 ns. The temperature was set to 310 K, and the pressure was maintained at 1 bar. Temperature and pressure were controlled using the V-rescale algorithm^54^ and the Parrinello–Rahman barostat,^55,56^ respectively. During this stage, a position-restraining force was applied to the heavy atoms, while the water and ions were allowed to move freely. After the equilibration stage, the constraints were removed, and the production runs were conducted in the NPT ensemble (T = 310 K, P = 1 bar) for 100 ns. The particle-mesh Ewald method^57^ was employed to treat long-range electrostatic interactions. The cutoff radii for both the Lennard-Jones potential and Coulomb forces were set to 1.2 nm. The LINCS algorithm^58^ was used to constrain all bonds involving hydrogen atoms, which allowed us to use a time step of 2 fs. All these steps were performed using GROMACS^53^ tools for preparing and running molecular dynamics simulations.

The Umbrella Sampling (US) technique was used to calculate the potential of mean force (PMF), which quantifies the binding strength between HSA and PET. Starting from the equilibrated configuration, HSA was pulled away from the PET surface using a force constant of 1000 kJ mol^–1^ nm^–2^. The center-to-center distance between HSA and the NP was chosen as the reaction coordinate for the PMF calculation. The reaction coordinate was divided into 17–24 windows, each spaced 2 Å apart. Each window was simulated for 20 ns, and the resulting structure was used as the starting configuration for the next window. Finally, the weighted histogram analysis method (WHAM)^59^ was applied to calculate the PMF. Error estimation of PMF profiles was performed using bootstrap resampling with 200 iterations, as implemented in the gmx wham tool from the GROMACS package. For each bootstrap sample, the PMF was recalculated using resampled force profiles from each umbrella window. The reported ΔG values correspond to the average of the bootstrapped PMFs, and the associated error bars represent the standard deviation across the bootstrap ensemble.

## 3 Results and Discussion

### 3.1 Degradation of PET Nanoparticles through Shock Compression in the Presence of Water

The analysis focuses on two PET nanoparticles of different sizes, either intact or subjected to increasing surface functionalization through shock compression in water. Table 1 lists the studied systems along with several important parameters associated with these systems.

**Table 1:** Systems Analyzed in This Study: S and L Denote Small and Large PET Nanoparticles, respectively, while the Number Indicates the Shock Compression Pressure Applied to a Given Nanoparticle^a^

Name	*P*, GPa	*T*_*eff*_, K	*R*_*g*_, nm	*N*_*Ow*_	*SASA, Å*^*2*^
S0	0		1.82	0	11701
S10	10	706	1.91	0	14265
S20	20	1208	1.89	22	14000
S25	25	1595	1.84	173	13121
S30	30	1865	1.80	284	12193
L0	0		2.32	0	18280
L10	10	706	2.34	0	23440
L20	20	1265	2.37	65	23674
L25	25	1530	2.36	194	21014
L30	30	1860	2.30	414	20680

a *P* represents the shock compression pressure, *T*_*eff*_ is the effective temperature to which the system is heated during the compression, *R*_*g*_ is the gyration radius, *N*_*Ow*_ is the number of oxygen atoms from water attached to the nanoparticle surface, and *SASA* is the solvent-accessible surface area of the nanoparticles.

As described in the Methods section, the initial structures of the PET nanoparticles (systems S0 and L0, Figure S1) were subjected to isotropic shock compression, with maximum pressure set to the values shown in Table 1. This process resulted in either purely mechanical compression of the systems or chemical reactions with water, as well as internal chemical transformations within the samples. As shown in Table 1, compression leads to an increase in the system’s temperature. The effective temperature (*T*_*eff*_) is reached within several picoseconds under the applied compression pressure and then remains constant. This creates extreme physical conditions within the simulation box, as both temperature and pressure reach very high values, resulting in the possibility of chemical transformations. These chemical reactions occur only when the compression pressure (and the associated effective temperature) exceed 10 GPa, manifesting as the addition of oxygen and hydrogen atoms from water to the surface of the PET nanoparticles. Figure 1 illustrates how the number of oxygen atoms and the mass of the nanoparticle increase over time.

As shown in Figure 1, shock compression causes a gradual increase in the number of oxygen atoms (*N*_*Ow*_) originating from water on the surface of PET nanoparticles. However, this addition does not occur at 10 GPa; only at 20 GPa and higher does the formation of surface oxygen groups from water become evident. Moreover, the higher the compression pressure, the more pronounced the increase.

The compression process (Hugoniostat dynamics) was run for 50 ps and then stopped, as indicated by the dashed vertical lines in Figure 1. The duration of the Hugoniostat dynamics was chosen somewhat arbitrarily, as extending it would have led to increasingly degraded surfaces and a higher number of surface oxygen groups, as shown in Figure 1. Afterward, the simulations were continued in a standard NPT ensemble to allow the systems to relax to 300 K. During equilibration, the number of surface oxygens originating from water and the masses of the nanoparticles stabilized at constant values, as observed in Figure 1. However, the nanoparticle surfaces revealed significant numbers of weakly bonded species, which would detach over longer time scales. To accelerate the detachment of these transient bonds, the systems were heated to 1000 K at constant volume. As shown in Figure 1, during heating, the number of added oxygen atoms and the masses of the nanoparticles decreased, eventually reaching new stabilized values, the mean values of which are summarized in Table 1. As shown in Table 1, a compression pressure of 20 GPa results in the formation of only 22 and 66 new surface oxygen groups for S and L nanoparticles, respectively. These values can be considered relatively small. In contrast, a compression pressure of 30 GPa leads to the formation of 284 and 414 new surface oxygen groups, which can be regarded as significantly high.

**Figure 2:**
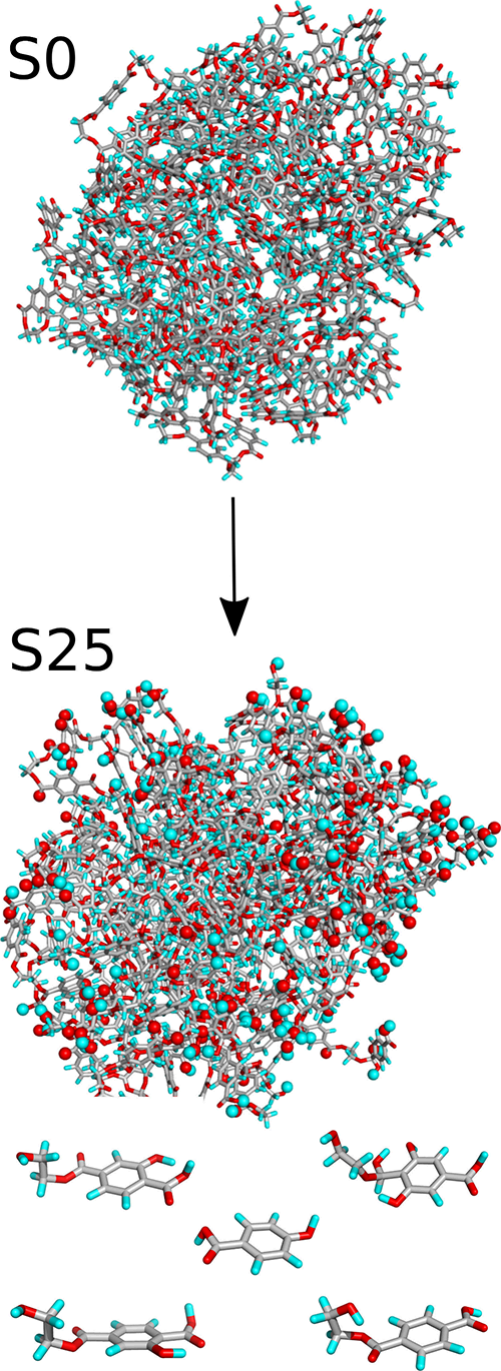
Visualization of the changes in the example PET nanoparticle structure after shock compression to 25 GPa. State S0 represents the unaltered nanoparticle before shock compression, while S25 depicts the nanoparticle after undergoing all treatments outlined in Figure 1 at a compression pressure of 25 GPa together with species detached from the main nanoparticle. The color coding is as follows: gray - carbon atoms, cyan - hydrogen atoms, and red - oxygen atoms. Oxygen and hydrogen atoms transferred from water and linked to the PET nanoparticle are represented as spheres.

A graphical representation of the changes occurring during shock compression is highly informative from a qualitative perspective. Figure 2 illustrates an example for the S25 case, where the initial S0 nanoparticle underwent all treatments outlined in Figure 1, reaching the final state denoted as S25. Along with the main nanoparticle, several other species also appeared, as shown at the bottom of Figure 2. These species detached from the nanoparticle and are present in the bulk solution. As shown in Figure 2 (or Figure S2 for other analyzed cases), the chemical structure of ethylene terephthalate, clearly visible in the S0 section of Figure 2, is significantly altered. The nanoparticle surface exhibits various functional groups that were previously absent. The oxygen and hydrogen atoms incorporated into the nanoparticle from water molecules are represented by spheres in the S25 section of Figure 2. Their substantial number, as indicated in Table 1, results in significant chemical modifications to the nanoparticle surface.

The bottom panel of Figure 1 illustrates how the molecular mass of the heaviest component evolves throughout the process. Initially, abrupt increases in mass are observed, attributed to the cross-linking of individual PET molecules into a single large molecule. This process is essentially irreversible, with the exception of the S nanoparticle at 10 GPa, where it temporarily fuses into a single molecule. In all other cases, including the L nanoparticle at 10 GPa, once fusion occurs, the molecules do not fully revert to their original state after the compression is halted.

The sizes of the nanoparticles, expressed as gyration radii in Table 1, have not changed significantly upon compression. However, small variations are observed, and their trends are not entirely straightforward. Lower compression pressures do not affect the gyration radii, but higher pressures, starting at 20 GPa, result in an increase in *R*_g_. This increase is attributed to the attachment of oxygen and hydrogen atoms from water. Conversely, even higher compression, such as 30 GPa, leads to a decrease in *R*_g_, which is due to the detachment of small molecular fragments from the nanoparticle caused by bond breaking under high compression pressure.

The surface structure of the nanoparticle, measured by the solvent-accessible surface area (SASA), behaves similarly to the *R*_g_. Specifically, a significant increase in SASA is observed when comparing untreated samples to those subjected to the lowest compression. This suggests that high-temperature treatment, while not inducing reactions with water, affected the surface structure, making it more open to the solvent. Higher compression levels lead to the development of an external surface due to the exposure of ″dangling″ molecular fragments (see Figure S2). At the highest pressure of 30 GPa, however, the external surface becomes less open due to the detachment of the most exposed fragments.

Thus, compression not only leads to the incorporation of oxygen and hydrogen atoms from water into the PET nanoparticle but also causes the release of these elements, originally part of the nanoparticle, into the surrounding bulk water. At higher compression pressures, larger molecular fragments may also detach from the PET nanoparticle. The compositions of the simulation boxes for all considered cases are summarized in Table S1 in the Supporting Information. Fragmentation of the nanoparticle begins at 20 GPa and becomes increasingly pronounced with higher compression pressures. The fragmentation products primarily consist of aromatic hydroxy carboxylic acids and esters containing 2 to 18 carbon atoms, accompanied by small quantities of ethene and carbon dioxide.

Chemical changes in the nanoparticles themselves are profound and depend primarily on the compression pressure. A detailed analysis of these changes becomes challenging at higher compression pressures due to the development of numerous cross-links within the nanoparticle skeletons, resulting in complex 3D chemical structures. Therefore, we primarily focused on the formation of new functional groups absent in the initial PET structure but significantly influencing the chemical properties of degraded PET nanoparticles. Table 2 presents a list of these functional groups, along with other notable structures that emerged after the degradation of PET nanoparticles through shock compression in the presence of water.

**Table 2:** Chemical Transformations of PET Nanoparticles as a Result of Shock Compression^a^

	S10	S20	S25	S30	L10	L20	L25	L30
ALT	8	247	1087	1852	36	664	1607	3079
EST	397	340	202	131	791	630	442	296
OH	0	25	141	231	0	85	209	357
CHO	0	1	10	11	0	3	6	19
COOH	0	0	21	25	0	6	19	42
CO	0	0	3	14	0	0	7	18
R3	0	0	5	5	0	0	4	6
R4	0	7	3	5	1	9	9	5
R5	2	9	8	16	3	13	24	25
R6	0	0	7	11	0	6	11	23

a The meaning of the factors in successive table rows is as follows: ALT refers to the number of atoms whose neighborhoods have been altered in any way; EST represents the number of ester groups; OH indicates the number of hydroxyl groups formed; CHO denotes the number of aldehyde groups formed; COOH corresponds to the number of carboxyl groups formed; CO refers to the number of carbonyl groups formed; R3 represents the number of 3-membered heterocyclic rings formed; R4 indicates the number of 4-membered heterocyclic rings formed; R5 denotes the number of 5-membered heterocyclic rings formed; R6 corresponds to the number of 6-membered heterocyclic rings formed.

As a general indicator of the degree of chemical transformation, we used the number of atoms whose surroundings changed in any way during the treatment outlined in Figure 1, referred to as ALT. Another general parameter indicating the degree of alteration in the chemical structure of the samples is the number of ester groups (EST) present in the ideal PET structure. The EST value is 200 for a single chain; thus, for a small nanoparticle, the EST value should be 400, while for a large nanoparticle, it should initially be 800.

**Figure 3:**
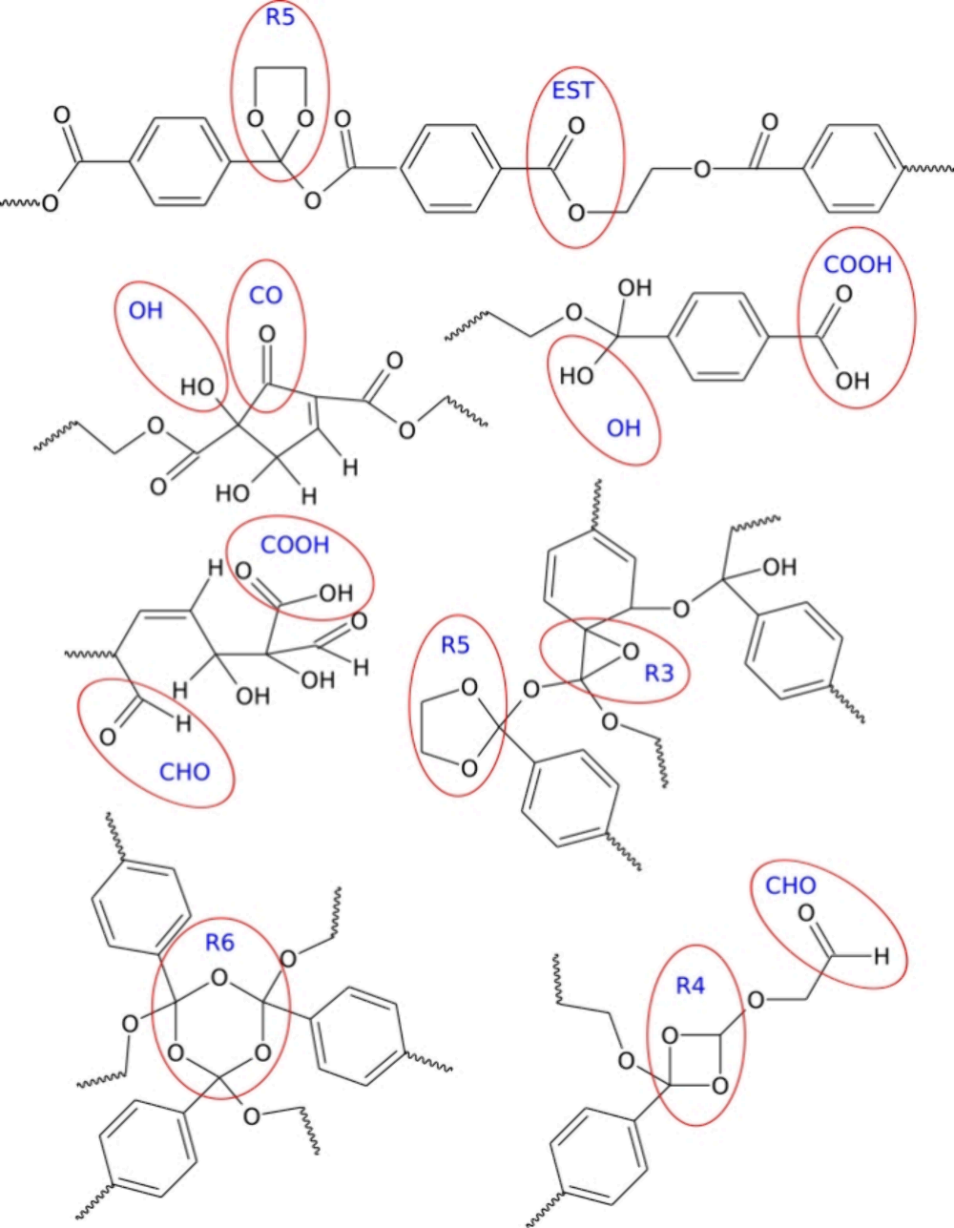
Selected structures found within the degraded PET nanoparticle, highlighting the presence of the functional groups listed in Table 2.

Examining changes in these two parameters with increasing compression pressure in Table 2, we observe that small changes occur even at a compression pressure of 10 GPa, which does not result in the attachment of any oxygen atoms from water. Similarly, EST slightly deviates from the reference value at 10 GPa, indicating that some irreversible alteration of the chemical structure of nanoparticles has occurred. This alteration begins with the formation of heterocyclic rings, R5 and R4, whose structural formulas are shown in Figure 3.

Figure 3 displays several selected structures identified within the degraded PET nanoparticles, which contain the functional groups listed in Table 2. These are not the only possible structures containing such groups but are rather common, as suggested by qualitative observations of the degraded nanoparticles.

Thus, as shown in Table 2, increasing compression pressure results in a significant increase in ALT and a corresponding decrease in EST. Quantitatively, approximately 50% of the atoms have a new surrounding at a compression pressure of 25 GPa, and half of the EST groups remain unaltered at this pressure. These values indicate significant degradation of the original structures. At a further compression pressure of 30 GPa, the level of structural destruction is so extensive that the resulting material can no longer be considered PET, but rather a completely different substance.

The populations of various functional groups, which develop primarily on the surface, reveal that hydroxyl groups are the most abundant. Their population increases rapidly, reaching almost 25% of the ester group count at a compression of 25 GPa. As shown in Figure 3, OH groups appear predominantly on aliphatic fragments of PET or within broken or dearomatized benzene rings. Direct attachment of OH groups to benzene rings was also observed but occurred less frequently.

Carboxyl groups are the second most common, although their population is only about 10% that of the OH groups. Carboxyl groups primarily form on benzene rings and typically require chain breaking for their appearance. Aldehyde (CHO) and carbonyl (CO) groups are significantly less frequent and mainly emerge from the breaking of benzene rings.

Thus, a general conclusion can be drawn that PET nanoparticle degradation in the presence of water predominantly results in the formation of hydroxyl groups on the surface, with oxygen atoms originating from either water or ester groups. A small amount of carboxyl groups is also expected, though their presence is more likely at higher levels of degradation.

The formation of various heterocyclic rings (R3-R6) primarily reflects transformations within the chemical structure of the PET nanoparticles’ bulk. These rings arise from bond rearrangements around ester groups or through fusion/cross-linking involving these structures. Five-membered rings seem to form more readily, while others require harsher conditions. The presence of such chemical species indicates significant alterations in the chemical properties of PET nanoparticles. However, these specific groups are typically not exposed on the surface but are instead confined to the bulk.

Among the six cases of surface-functionalized PET nanoparticles studied, we selected two for further investigation of their interactions with human serum albumin (HSA). These are the S25 and L25 nanoparticles, as they exhibit significant levels of degradation while still retaining the fundamental chemical nature of PET.

### 3.2 Interaction of PET Nanoparticles with Human Serum Albumin

To investigate how the degradation of PET nanoparticles affects their interaction with biologically important molecules, we selected human serum albumin (HSA) as a case study. The calculations were performed using the standard structure of HSA obtained from the Protein Data Bank (PDB ID^45^) and four types of PET nanoparticles. These include two ideal, nondegraded nanoparticles of different sizes—small (S0) and large (L0)—as well as two degraded nanoparticles, S25 and L25, with all relevant parameters described in Tables 1 and 2. The molecular and force field topology files of S25 and L25 in GROMACS format are available in the Supporting Information section.

As described in the Methods section, finding the optimal configuration was based on rolling the PET nanoparticles on the surface of HSA using a simplified mechanistic model, i.e., an implicit solvent and rigid body motion.^44^ As shown in Figure S4, the trace of the PET nanoparticle during the scanning covers almost the entire surface of HSA. There are also areas where the PET molecules were avoided or spent very little time. These correspond to configurations where the interaction energy between HSA and PET was low or close to zero, meaning that such spatial arrangements of these two objects are highly energetically unfavorable. These energies are shown in Figure S3 as functions of time, along with the highlighted regions of the configurational space from which the full all-atom simulations with water were initiated. After 50 ns of equilibration in the NPT ensemble, each system was run for an additional 100 ns for production. Thus, in the following discussion, we use the trajectories obtained from these production runs for analysis.

**Figure 4:**
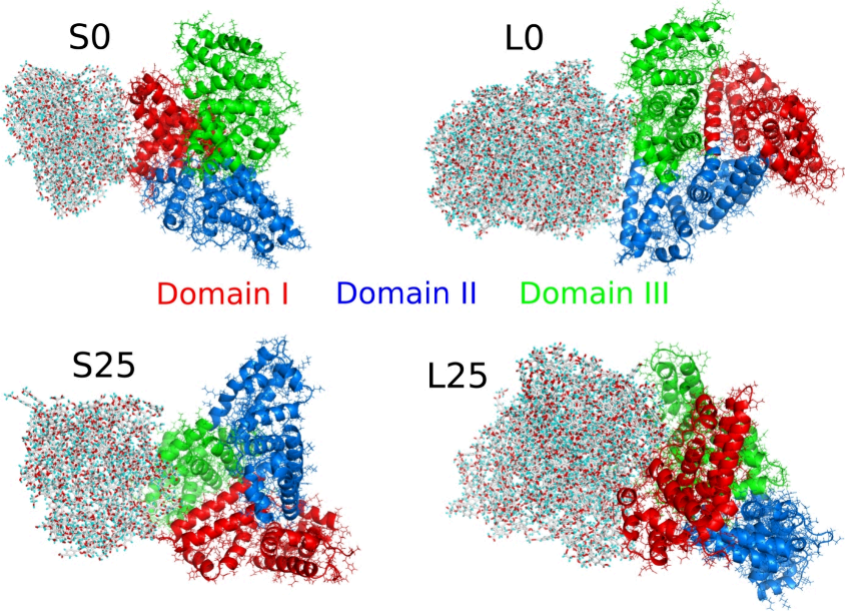
Binding configurations of PET-HSA for various PET nanoparticles. S0 and L0 refer to nondegraded, ideal PET nanoparticles, small and large, respectively, according to the nomenclature from Table 1. S25 and L25 correspond to degraded small and large PET nanoparticles obtained after shock compression and treatment as outlined in Figure 1. HSA is represented as a cartoon with its three domains depicted in different colors: red for domain I, blue for domain II, and green for domain III.

Figure 4 shows the detected binding configurations of PET on the HSA surface. As is well-known, HSA can be divided into three domains, each playing a specific role. Domain I is involved in binding metal ions and contributes to stabilizing proteins and other interacting ligands. Domain II is crucial for the transport of drugs and fatty acids, as well as for maintaining acid–base balance. Domain III, in turn, binds hydrophobic moieties and various drugs and is responsible for the antioxidant properties of HSA. Given these functional roles, identifying the binding sites of PET nanoparticles on the HSA surface is both scientifically interesting and biologically significant.

As seen in Figure 4, the S0 PET nanoparticle binds almost exclusively to Domain I of HSA, which is also confirmed by the number of close contacts collected in Table 3. A small fraction of close contacts (12%) is associated with Domain II, indicating that the nanoparticle occasionally interacts with this part of the protein. The large but nondegraded L0 nanoparticle binds to both Domain II and Domain III, with a slight predominance of contacts in Domain III. Interestingly, the degradation of these two nanoparticles leads to PET nanoparticles binding to both Domain I and Domain III in roughly equal proportions.

**Table 3:** Pair Interaction Energies and the Number of Close Contacts Determined for the Equilibrium Configurations of HSA-PET, as Shown in Figure 4^a^

	Pair energy, kJ mol^–1^							
	HSA-PET		PET-water		Number of close contacts			
System	Coul	vdW	Coul	vdW	total	domain I	domain II	domain III
S0	–68.6 ± 33.5	–316.4 ± 32.1	–6384 ± 205	–3447 ± 91	301.6 ± 19.1	88%	12%	0
L0	–88.0 ± 38.3	–404.2 ± 34.7	–13257 ± 279	–6313 ± 139	364.6 ± 27.7	0	42%	58%
S25	–126.5 ± 70.3	–162.7 ± 39.2	–16398 ± 311	–3586 ± 128	257.3 ± 39.5	46%	0	54%
L25	–210.7 ± 55.8	–382.6 ± 30.5	–31251 ± 431	–7204 ± 174	437.8 ± 22.8	52	0	48%

a The PET-water interaction energies were calculated in separate simulations without HSA in the simulation boxes.

The total number of close contacts remains relatively stable, as shown in Figure S5, suggesting that the binding sites are well recognized by our search procedure. An intriguing observation is that for large nanoparticles, degradation increases the total number of close contacts, whereas for small nanoparticles, degradation reduces the total number of close contacts. Additionally, fluctuations in this factor are the highest for the S25 nanoparticles, indicating that binding in this case is the weakest.

**Figure 5:**
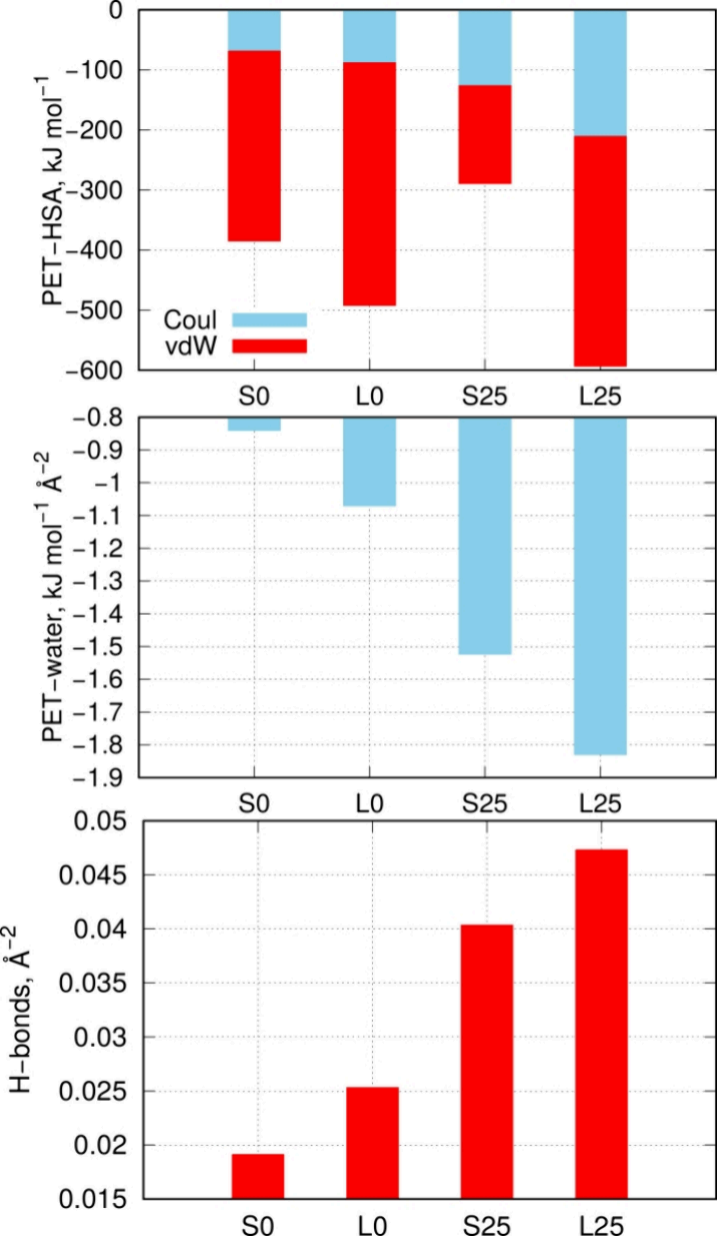
(Upper part) Pair interaction energies between PET and HSA, split into the electrostatic component (Coul) and the dispersion interaction component (vdW). (Middle part) PET-water interaction energy recalculated per unit of Solvent Accessible Surface Area (SASA). (Bottom part) Number of hydrogen bonds per unit of Solvent Accessible Surface Area determined for free, noninteracting with HSA, PET nanoparticles.

The binding strength is mainly controlled by the pair interaction energy between atoms from HSA and PET. This component is presented in Table 3 and illustrated graphically in Figure 5. As expected, the pair interaction energy is greater for larger nanoparticles due to the higher number of interacting pairs. We can also observe that the primary component of the energy is the van der Waals interaction. However, degradation leads to significant changes in the proportions of energy components. For large nanoparticles, the van der Waals interaction remains at a similar level for both intact and degraded nanoparticles, but the electrostatic component increases significantly for the degraded nanoparticle (L25). Similarly, for small nanoparticles, the electrostatic component increases upon degradation, while the van der Waals component decreases substantially. This results in an overall reduction of the pair interaction energy for the degraded small nanoparticle (S25) compared to the intact one (S0).

Another important factor controlling the state of the HSA-PET configuration is the interaction with water. Since only the PET nanoparticles undergo changes in their surface properties, we determined the pair interaction energies of the PET nanoparticles with water in the absence of HSA to analyze how degradation affects this energy. As shown in Table 3, the primary energy component is the electrostatic interaction, which changes significantly with the state of the nanoparticle, while the van der Waals component remains nearly constant. The strong increase in both the pair interaction energy with water and the electrostatic energy component upon degradation indicates that the degraded nanoparticles become more hydrophilic.

The pair interaction energy with water, when recalculated per unit of solvent-accessible surface area, serves as a quantitative measure of hydrophilicity, as illustrated in Figure 5. Thus, we can draw a clear conclusion that degradation leads to a significant increase in nanoparticle hydrophilicity. In the case of the small PET nanoparticle, hydrophilicity increased more than 10-fold upon degradation, while for the large one, the increase was at least 4-fold. This is consistent with the data from Table 2, which shows a large population of hydroxyl groups forming as a result of PET nanoparticle degradation in the presence of water.

An additional factor confirming the significant increase in nanoparticle hydrophilicity upon degradation is the number of hydrogen bonds they form with water. As shown in Figure 5, for small nanoparticles, degradation led to more than a 2-fold increase in the number of hydrogen bonds per square angstrom compared to the initial, nondegraded S0 nanoparticle. For large nanoparticles, the increase is also substantial, though slightly less intense, as it is less than double compared to the L0 nanoparticle.

An important factor that may result from the interaction of PET nanoparticles with HSA is the modification of the protein’s internal structure, including its secondary structure. A commonly used parameter for analyzing structural changes in proteins is the root mean squared deviation (RMSD) from a reference state. RMSD is a measure used to quantify the difference between the positions of atoms in two structures: the studied one and a reference structure. In this case, we used the HSA structure equilibrated in water as the reference.

**Figure 6:**
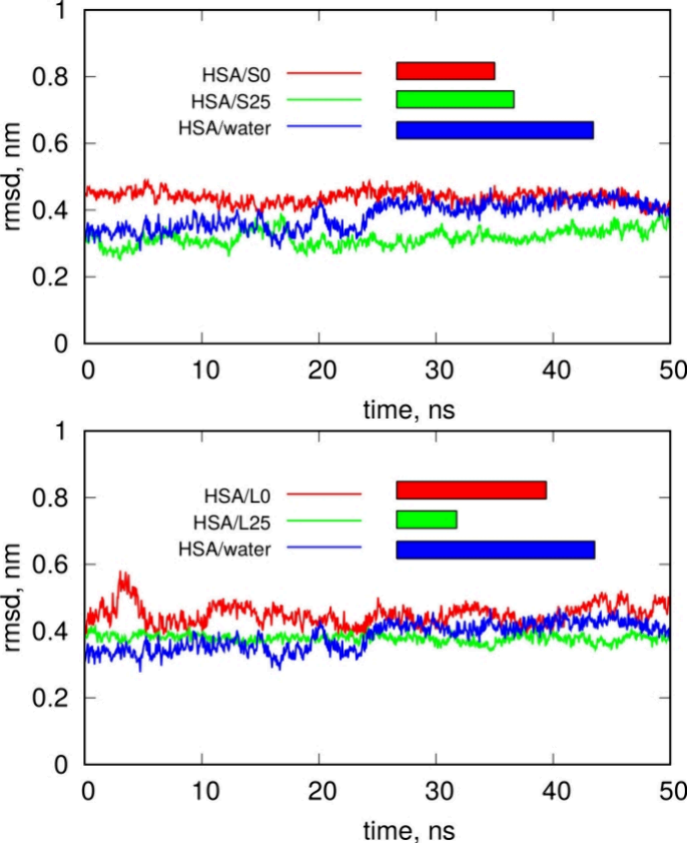
Root mean squared displacement (RMSD) of the HSA structure upon interaction with degraded and intact PET nanoparticles. The upper part corresponds to small PET nanoparticles, while the bottom part represents large PET nanoparticles. The lengths of the color bars next to the key illustrate the relative standard deviations of RMSD for each case. The values of standard deviations are following: HSA/water 0.041 nm; HSA/S0 0.021 nm; HSA/S25 0.025 nm; HSA/L0 0.031 nm and HSA/L25 0.0127 nm.

Figure 6 shows the RMSD plots of HSA determined over the last 50 ns of simulations, either for a free HSA molecule in water or in cases where HSA interacts with PET nanoparticles. The RMSD plots in Figure 6 do not reveal any drifts indicative of unfolding or other structural transformations of HSA. On the contrary, the plots remain practically stable, and the RMSD values are typical of thermal fluctuations in a stable structure. The lengths of the colored bars in Figure 6 represent the relative standard deviations of RMSD for each case. We can observe that the largest fluctuations occur in HSA without PET, whereas the presence of PET nanoparticles interacting with HSA leads to structural stabilization. Small PET nanoparticles stabilize HSA at a similar level, regardless of whether the particle is degraded or intact. In the case of large nanoparticles, the degraded PET nanoparticle provides the most noticeable stabilization of the HSA structure.

**Figure 7:**
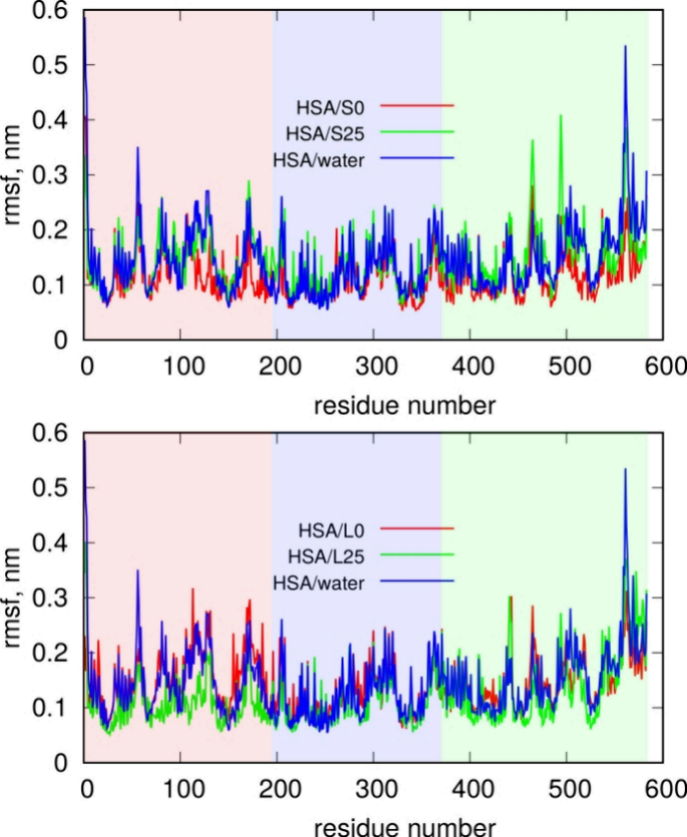
Root mean squared fluctuation (RMSF) of HSA interacting with degraded and nondegraded PET nanoparticles. The upper part corresponds to small PET nanoparticles, while the bottom part represents large PET nanoparticles. The red, blue, and green backgrounds on the graphs indicate residues belonging to domain I, domain II, and domain III of HSA, respectively.

Similar conclusions can be drawn from the analysis of the root mean squared fluctuation (RMSF) of subsequent residues in the HSA protein. RMSF is a measure used to quantify the fluctuations or variability of atomic positions in a molecule during a molecular dynamics simulation, relative to a reference structure, which in this case is the HSA structure equilibrated in water. Figure 7 shows the plot of this quantity, with the marked ranges of residues belonging to domains I–III. For small, nondegraded nanoparticles, we observe a reduction in fluctuations within domains I and III compared to free HSA. However, the degraded small PET nanoparticle slightly increases fluctuations within domain III. In the case of the degraded large nanoparticle (S25), a noticeable reduction in fluctuations within domains I and III can be observed. Nevertheless, none of these changes in RMSF plots indicate significant structural transformations of the HSA protein upon interaction with PET nanoparticles.

**Figure 8:**
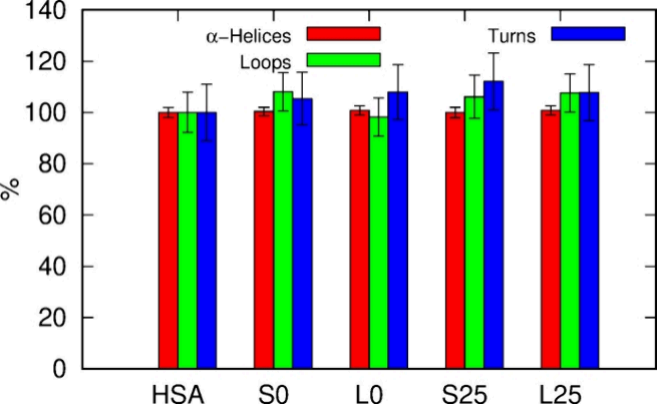
Percentage of amino acid residues in α-Helices, Loops, and Turns relative to pure HSA, determined for PET–HSA conjugates.

A more detailed analysis of the HSA structure was conducted by performing a statistical assessment of the number of residues belonging to α-Helices and Loops. This secondary structure analysis is presented in Figure 8 as the percentage of a given secondary structure type relative to that of pure HSA. Free HSA contains approximately 390 amino acid residues forming α-Helices, 58 belonging to Loops, and no amino acids forming β-Sheets. Upon interaction with a ligand, the protein may undergo structural transformations, leading to a different distribution of amino acids among secondary structure elements. However, as shown in Figure 8, the interaction of HSA with PET nanoparticles, whether intact or degraded, does not alter the secondary structure of the HSA protein. It is clearly seen that the amount of α-Helices remains unchanged upon interaction with PET, and the corresponding standard deviations are small. Other structures, such as Loops and Turns, show a slight increase compared to pure HSA, but this does not exceed 10%. Considering that the amounts of Loops and Turns fluctuate significantly both for pure HSA and in interaction with PET, we conclude that these forms of the protein’s secondary structure are also only marginally affected by the presence of PET. This finding is somewhat surprising, given the strong interaction between HSA and PET nanoparticles, especially considering literature reports highlighting protein unfolding upon interaction with polystyrene nanoparticles.^18^ However, it appears that the interaction is strongly size-dependent, which may explain the reported unfolding of HSA when interacting with polystyrene nanoparticles with a large diameter of 100 nm. In our case, the nanoparticles have sizes comparable to HSA and are significantly smaller than 10 nm in diameter.

Special attention has also been given to the structure of the hydrophobic drug binding sites of HSA, localized around tryptophan residues TRP214 and TRP218, which also play a significant role in interactions with aromatic ligands through π-π stacking interactions. Our analysis led to the conclusion that no significant alterations in these structures are observed upon interaction with PET nanoparticles. Their RMSF values range from 0.058 to 0.088 nm across all studied cases, i.e., HSA alone and in contact with PET nanoparticles.

**Figure 9:**
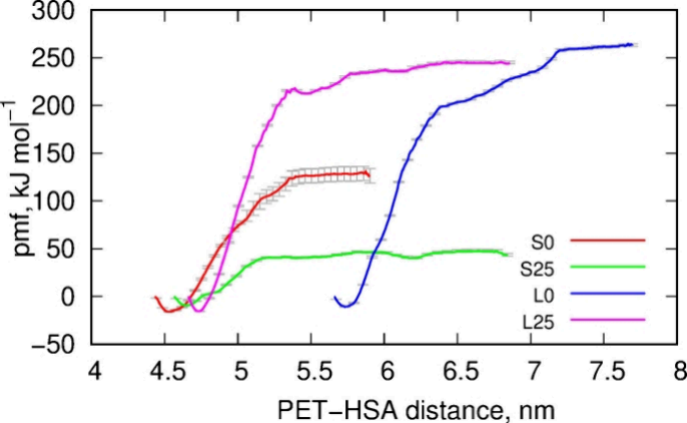
Potentials of mean force determined during umbrella sampling simulations and weighted histogram analysis for the desorption of PET nanoparticles from HSA surfaces. The ΔG values for adsorption can be determined as the heights of the PMF curves during the separation of these species, taken with reversed signs. Thus, for S0, ΔG = – 145 kJ mol^–1^; for S25, ΔG = – 55 kJ mol^–1^; for L0, ΔG = – 272 kJ mol^–1^; and for L25, ΔG = – 260 kJ mol^–1^. Error estimation of PMF profiles was performed using bootstrap resampling with 200 iterations, as implemented in the gmx wham tool from the GROMACS package.

The thermodynamic state of the PET-HSA conjugates was studied using umbrella sampling calculations, which enforced the detachment of PET nanoparticles from HSA. As mentioned in the Methods section, the starting configurations for umbrella sampling were those shown in Figure 4, corresponding to the strongest interacting arrangements of these two species. According to the formal interpretation of the difference in potentials of mean force, obtained from the weighted histogram analysis, this difference can be identified with the thermodynamic free energy of adsorption ΔG. The plots of the potentials of mean force for each system, obtained from the weighted histogram analysis, are shown in Figure 9.

Analysis of the free energy values, presented in the caption to Figure 9, leads to conclusions consistent with previous analyses of pair interaction energies and the numbers of close contacts. Specifically, large nanoparticles, whether degraded or not, strongly adsorb/bind to HSA. The process is essentially irreversible, as the free energies are approximately −250 kJ mol^–1^. Indeed, we observed very stable PET-HSA conjugates in these cases, and the likelihood of PET nanoparticles spontaneously detaching from HSA is very low. Degradation, which led to significant alterations in the surface chemistry of the nanoparticles, had a relatively small effect, as the difference in free energies between degraded and intact nanoparticles is only 12 kJ mol^–1^, indicating that degradation slightly weakens adsorption.

Contrary to large nanoparticles, the small ones exhibited quite surprising behavior. As shown in Figure 9, intact nanoparticles bind to HSA with a still high free energy of adsorption ΔG = – 145 kJ mol^–1^, indicating a stable and essentially irreversible state. However, the degraded small nanoparticle (S25) showed a significant reduction in the free energy of adsorption ΔG = – 55 kJ mol^–1^, suggesting weak adsorption with a high probability of desorption due to thermal fluctuations alone. Formally, the process remains spontaneous, but the free energy well is too shallow to ensure stable binding of these two species in solution.

The molecular explanation for these observations is relatively straightforward. Degradation led to a significant increase in the hydrophilicity of PET nanoparticles, as shown in Figure 5, due to the formation of numerous hydrophilic surface groups (hydroxyl, carboxyl, aldehyde). This weakened adsorption to HSA because interactions with water became more favorable. Large nanoparticles have a smaller surface-to-volume ratio, resulting in only a slight weakening of the free energy of adsorption. In contrast, small nanoparticles have a much larger surface-to-volume ratio, and as a result, interactions with water became dominant (or nearly dominant) over interactions with HSA, making the separated state more favorable. An analogous effect of hydrophilicity on the adsorption of HSA on graphene or gold surfaces has been described in the literature. Increasing hydrophilicity by augmenting the number of surface hydroxyl groups leads to a reduction in the interaction energy between HSA and the surface.^60,61^

We can thus draw a general conclusion that the binding of degraded PET nanoparticles to HSA may be strong or weak, depending on the size of the PET nanoparticle. Additionally, the shapes of both the protein and the nanoparticles can affect the binding energy, albeit in a rather nonsystematic manner, as shown in ref (61). Nanoparticles with sizes comparable to or larger than HSA will bind strongly, whereas smaller nanoparticles, roughly half the size of HSA, will bind weakly or may not bind at all in aqueous solution. Assessing which scenario is more concerning in terms of the potential toxicity of degraded PET nanoparticles is challenging. It is well-known that one of the important functions of HSA is the transport and clearance of various substances and metabolic byproducts from the bloodstream. Therefore, large nanoparticles are more likely to be captured by HSA and neutralized through metabolic pathways. In contrast, small degraded PET nanoparticles, which are not effectively trapped by HSA, may migrate freely in the bloodstream, posing a risk to various cellular processes or even interacting with DNA.

## 4 Summary and Conclusions

This study investigates the interactions between degraded polyethylene terephthalate nanoparticles and human serum albumin, emphasizing the effects of nanoparticle size and surface modifications due to degradation. PET degradation was induced using shock compression in water, leading to significant chemical transformations, including the formation of hydroxyl, carboxyl, and carbonyl groups on the nanoparticle surfaces. These modifications influenced the hydrophilicity and binding behavior of PET nanoparticles with HSA.

The study utilized molecular dynamics simulations, umbrella sampling, and weighted histogram analysis to explore the thermodynamic aspects of PET-HSA interactions. The binding configurations of PET nanoparticles on HSA were determined, revealing that degraded PET nanoparticles preferentially bind to Domain I and Domain III of HSA, while nondegraded nanoparticles exhibited changes in binding affinity and site preference. The interaction energy analysis indicated that larger PET nanoparticles exhibited stronger binding, whereas small degraded PET nanoparticles had significantly reduced interaction energies, suggesting a higher probability of desorption.

Analysis of root mean squared deviation (RMSD) and root mean squared fluctuation (RMSF) confirmed that PET binding does not induce significant structural changes in HSA. The protein maintains its stability, with small fluctuations observed depending on nanoparticle type. Notably, degradation led to an increase in hydrophilicity, as confirmed by interaction energy calculations with water, with small PET nanoparticles exhibiting a 10-fold increase in hydrophilic interactions upon degradation. This shift in affinity toward water weakened their adsorption onto HSA, making detachment more probable.

We can draw several specific conclusions from this study: *Effect of Degradation on Binding Strength:* Large PET nanoparticles, whether degraded or not, strongly adsorb onto HSA, forming stable and nearly irreversible complexes. In contrast, small degraded nanoparticles exhibit weak adsorption and a high likelihood of desorption due to thermal fluctuations. *Size-Dependent Binding Behavior:* PET nanoparticles of comparable size or larger than HSA maintain strong binding interactions. However, small PET nanoparticles, approximately half the size of HSA, tend to exhibit weak or negligible binding. *Hydrophilicity Influence:* Degradation significantly increases the hydrophilicity of PET nanoparticles, particularly for smaller ones, making their interactions with water stronger than with HSA. This reduces their ability to form stable conjugates with HSA in solution. *Biological Implications:* While large degraded nanoparticles are likely to be captured by HSA and processed through metabolic pathways, small degraded nanoparticles remain free in the bloodstream. This raises concerns about their potential toxicity, as they may migrate and interfere with cellular functions or even interact with DNA. *Minimal Structural Impact on HSA:* Despite strong interactions, PET nanoparticles do not significantly alter the secondary structure of HSA. The protein remains stable, showing only minor fluctuations upon binding.

## Supporting Information Available

Visualizations of the simulation boxes with the smaller/larger PET nanoparticles and after shock compression and subsequent treatments, components of the simulation boxes after performing shock compression, results of exploring the configurational phase space, snapshots highlighting the regions of HSA, parameters of the simulation boxes, and number of close contacts (PDF)
Atomic and force field topology data in GROMACS format for the degraded L25 GMX PET nanoparticle (TXT)
Atomic and force field topology data in GROMACS format for the degraded L25 gro PET nanoparticle (TXT)
Atomic and force field topology data in GROMACS format for the degraded S25 gro PET nanoparticle (TXT)
Atomic and force field topology data in GROMACS format for the degraded S25 GMX PET nanoparticle (TXT)
