# Supplementary material for: Size-Dependent Interactions of Degraded PET Nanoparticles with Human Serum Albumin: Thermodynamic and Molecular Insights
Source: J Phys Chem B. 2025 Apr 28;129(18):4581–94. doi: 10.1021/acs.jpcb.5c01362 (PMC12067432; doi:10.1021/acs.jpcb.5c01362)
Supplement: Supplementary file 1 — jp5c01362_si_001.pdf [file jp5c01362_si_001.pdf]

Size-Dependent Interactions of Degraded PET Nanoparticles with Human Serum Albumin: Thermodynamic and Molecular Insights

Tomasz Panczyk[1,*], Pawel Wolski[1] and Krzysztof Nieszporek[2]

[1]Jerzy Haber Institute of Catalysis and Surface Chemistry, Polish Academy of Sciences ul. Niezapominajek 8,30239 Cracow, Poland
[2]Department of Theoretical Chemistry, Institute of Chemical Sciences, Faculty of Chemistry, Maria Curie-Sklodowska University in Lublin pl. Maria Curie-Sklodowska 3, 20031 Lublin, Poland
*Correspondence: tomasz.panczyk@ikifp.edu.pl

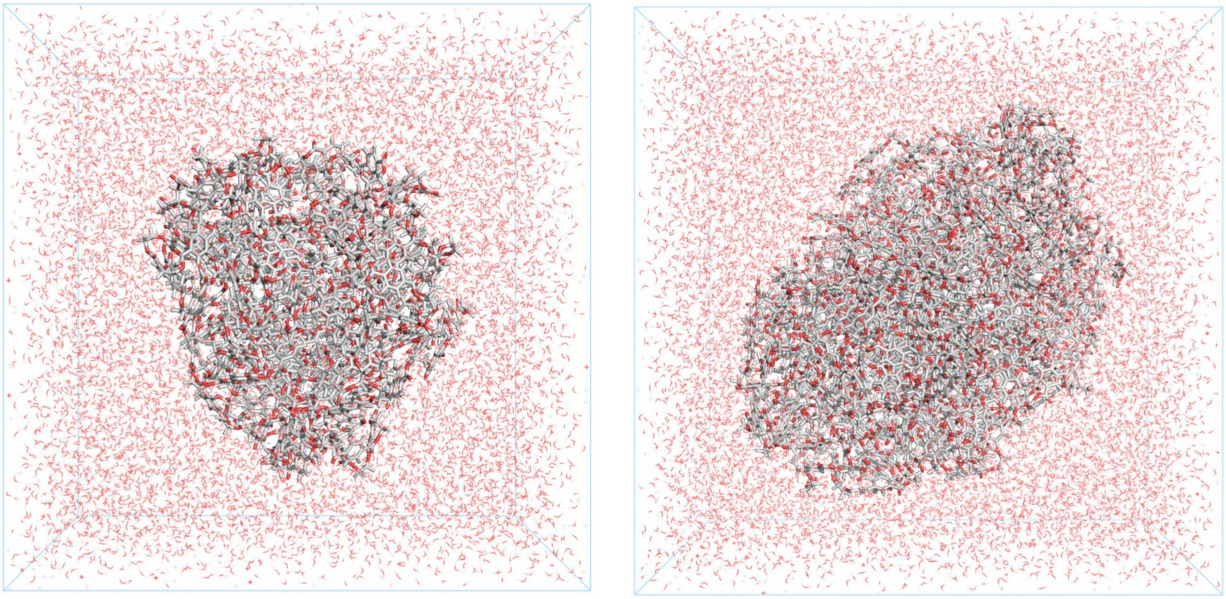

Figure S1. Visualizations of the simulation boxes with the smaller PET nanoparticle S (left) and the larger PET nanoparticle L (right) after equilibration in the NPT ensemble using the ReaxFF force field. The temperature and pressure were maintained at 300 K and 1 bar, respectively. The sizes of the nanoparticles, expressed as gyration radii, are 1.82 nm and 2.31 nm, depending on the initial number of PET chains used.

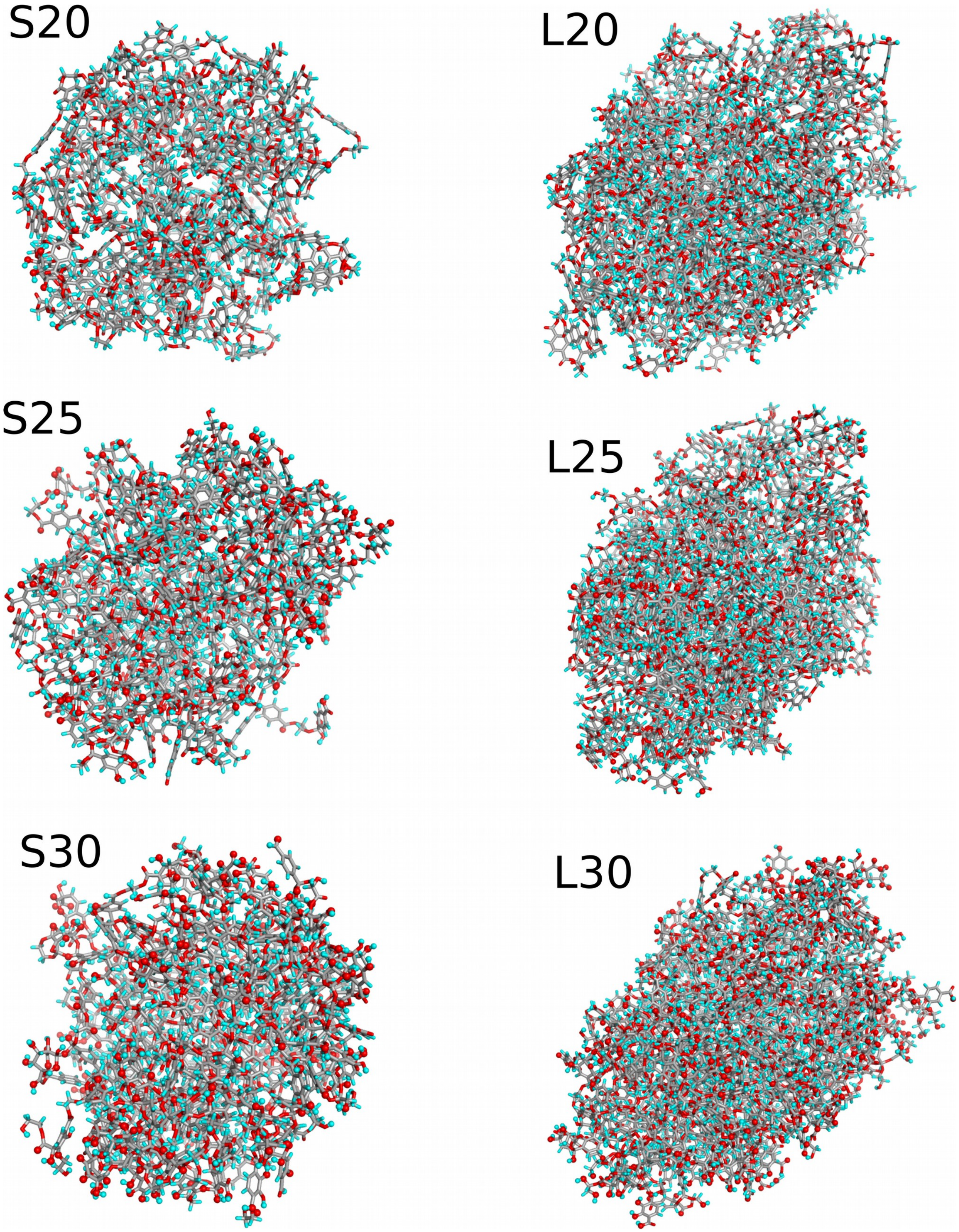

Figure S2. Visualizations of PET nanoparticles after shock compression and subsequent treatments, as outlined in Fig. 1. The color coding is as follows: gray represents carbon atoms, cyan represents

hydrogen atoms, and red represents oxygen atoms. Oxygen and hydrogen atoms transferred from water are depicted as spheres.

Table S1. Components of the simulation boxes after performing shock compression of PET nanoparticles in water. The blue fragments in the main component formulas indicate the oxygen and hydrogen atoms derived from water.

Compression pressure, GPa	Main component	Other components
Small nanoparticle		
S10	2 × $C_{1002}H_{806}O_{400}$	-
S20	$C_{2004}H_{1606}O_{800}\mathit{O_{22}H_{24}}$	-
S25	$C_{1957}H_{1490}O_{757}\mathit{O_{173}H_{146}}$	$C_{10}H_8O_3O_3H_2$, $C_{10}H_9O_4OH$, $C_{10}H_6O_3O_4H_6$, $C_{10}H_7O_5OH_3$, $C_7H_4O_3H_2$
S30	$C_{1984}H_{1365}O_{747}O_{284}H_{257}$	$C_2H_4O_2H_2$, C_2H_5OOH, $C_2HO_2O_2H_3$, $C_2H_3O_2H$, $C_2H_3O_3H_3$, C_2H_4, $C_2H_4OOH_2$, CH_3OH, CO_2
Large nanoparticle		
L10	$C_{2004}H_{1612}O_{800}$ + 2 × $C_{1002}H_{806}O_{400}$	-
L20	$C_{3999}H_{3201}O_{1592}\mathit{O_{65}H_{84}}$	$C_9H_7O_3OH$
L25	$C_{3973}H_{3096}O_{1561}\mathit{O_{194}H_{202}}$	$C_{12}H_{11}O_6OH_3$, $C_{11}H_{10}O_5OH_2$, C_3H_7OH, C_2H_3OOH, CH_3OH, C_2H_4
L30	$C_{3932}H_{2790}O_{1486}\mathit{O_{414}H_{394}}$	$C_{18}H_8O_6O_7H_7$, $C_{14}H_{14}O_6O_3H_6$, $C_8H_3OO_4H_3$, C_3H_8O, $C_2H_3OO_2H_3$, $C_2H_4OOH_2$, $C_2H_4O_2H_2$, $C_2H_4O_2$, C_2H_3OH, C_2H_3OOH, C_2H_4

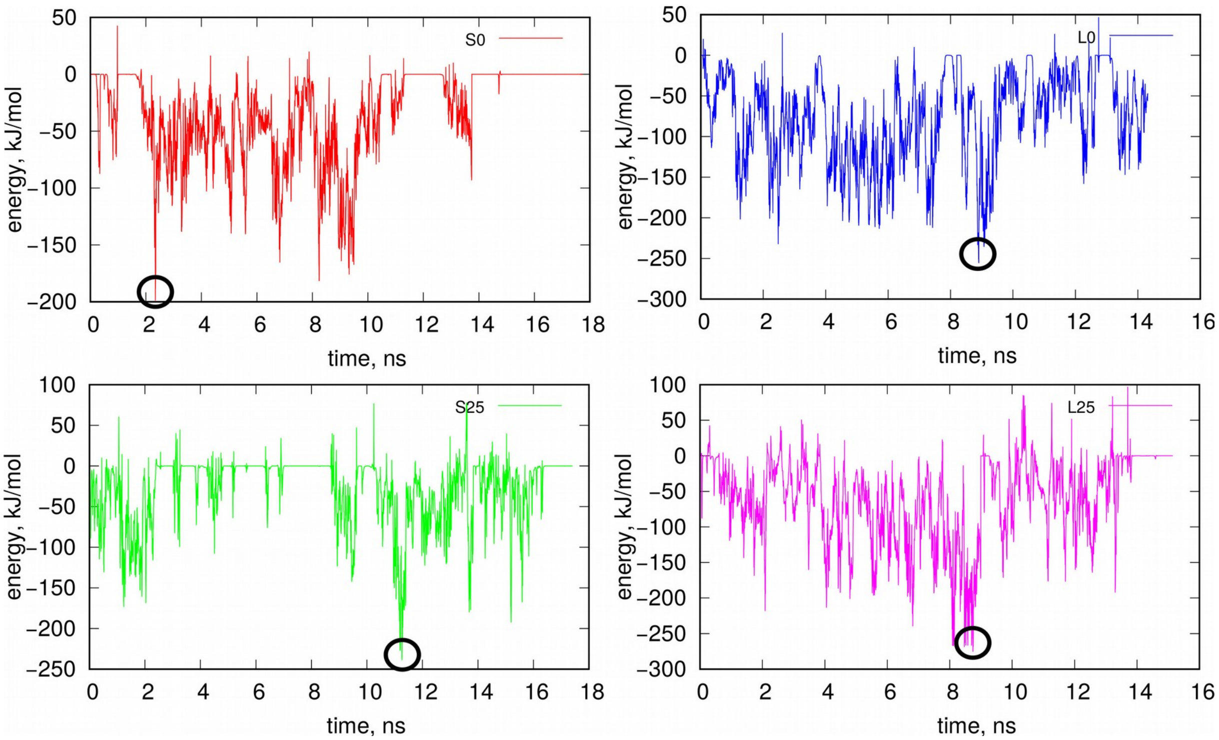

Figure S3. Results of exploring the configurational phase space of PET-HSA interactions using rigid-body implicit solvent simulations. The configurations were generated through metadynamics applied to the polar angle, measured from the z-axis relative to the HSA molecule's center of mass, and the azimuthal angle of its orthogonal projection onto the x–y plane. The black circles indicate the configurations with the best rigid-body matching, which served as starting points for full simulations incorporating flexibility and explicit water dynamics.

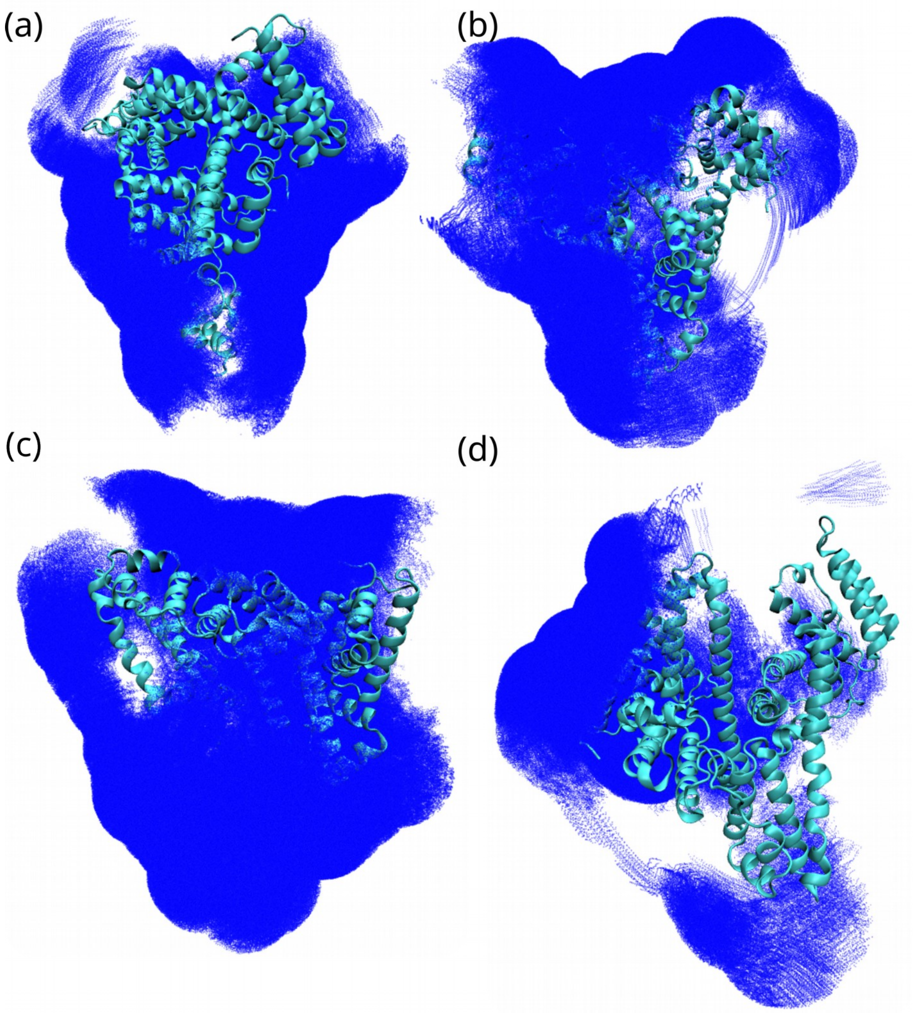

Figure S4. Snapshots highlighting the regions of HSA (blue points) visited by the PET nanoparticle during the scanning procedure. (a) L25, (b) S25, (c) L0, and (d) S0. The blue points represent the atoms of the PET nanoparticle that were within a 12 Å distance (i.e., the interaction cutoff distance) from any HSA atom. The HSA protein is shown in a cartoon representation.

Table S2. Parameters of the simulation boxes for simulations of the interactions between PET and HSA, and for umbrella sampling calculations of the potential of mean force.

System	Standard simulations				Umbrella sampling			
	Box size (nm)	No. of water	No. of Na^+	No. of Cl^-	Box size (nm)	No. of water	No. of Na^+	No. of Cl^-
L25	14x14x14	85331	261	253	19x11x11	68368	216	208
S25	13x13x13	65973	205	197	17x11x11	61992	194	186
L0	15.5x15.5x15.5	119650	357	349	20x11x11	72453	227	219
S0	13.5x13.5x13.5	76740	232	224	18x11x11	66094	205	197

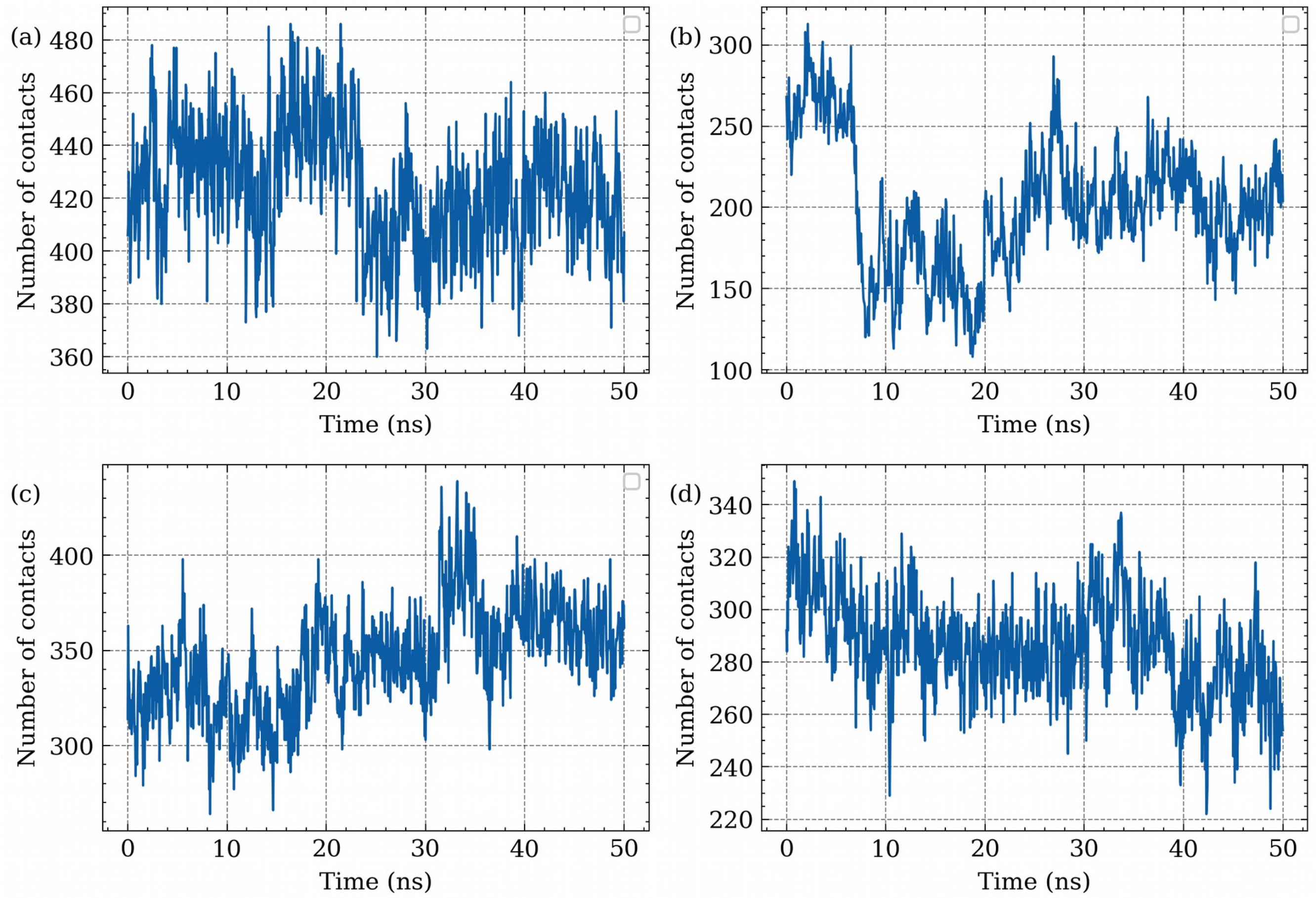

Figure S5. The number of close contacts (i.e., distances less than 0.6 nm) for a given pair of atoms belonging to HSA and PET as a function of time for the studied HSA-PET systems: (a) L25, (b) S25, (c) L20, and (d) S20.
